# Effect of Inoculum Pretreatment on the Composition of Microbial Communities in Anaerobic Digesters Producing Volatile Fatty Acids

**DOI:** 10.3390/microorganisms8040581

**Published:** 2020-04-17

**Authors:** Lucia Blasco, Minna Kahala, Elina Tampio, Markku Vainio, Satu Ervasti, Saija Rasi

**Affiliations:** Natural Resources Institute Finland (Luke), Production Systems, Tietotie 4, FI-31600 Jokioinen, Finland; lucia.blasco@luke.fi (L.B.); minna.kahala@luke.fi (M.K.); markku.vainio@luke.fi (M.V.); satu.ervasti@luke.fi (S.E.); saija.rasi@luke.fi (S.R.)

**Keywords:** microbial communities, volatile fatty acids, anaerobic digestion, 16S rDNA amplicon sequencing, methanogenic archaea, biowaste

## Abstract

Volatile fatty acids (VFAs) are intermediates in the methane formation pathway of anaerobic digestion and can be produced through the fermentation of organic wastes. VFAs have become an anticipated resource- and cost-effective way to replace fossil resources with higher added value and more versatile fuels and chemicals. However, there are still challenges in the production of targeted compounds from diverse and complex biomasses, such as urban biowastes. In this study, the aim was to modulate the microbial communities through inoculum treatment to enhance the production of green chemicals. Thermal and freeze-thaw treatments were applied to the anaerobic digester inoculum to inhibit the growth of methanogens and to enhance the performance of acidogenic and acetogenic bacteria. VFA fermentation after different inoculum treatments was studied in batch scale using urban biowaste as the substrate and the process performance was assessed with chemical and microbial analyses. Inoculum treatments, especially thermal treatment, were shown to increase VFA yields, which were also correlating with the dynamics of the microbial communities and retention times of the test. There was a strong correlation between VFA production and the relative abundances of the microbial orders Clostridiales (families *Ruminococcaceae*, *Lachnospiraceae* and *Clostridiaceae*), and Lactobacillales. A syntrophic relationship of these taxa with members of the Methanobacteriales order was also presumed.

## 1. Introduction

To enable the recycling of organic carbon in the society, the valorization of organic biomasses, such as urban biowastes (BW), is essential. BWs are often valorized through anaerobic digestion, which facilitates simultaneous production of renewable energy in the form of methane and production of nutrient-rich residue, digestate. Anaerobic digestion is a synergistic process of mixed bacteria and archaea, where hydrolysis, acidogenesis, acetogenesis and methanogenesis occur simultaneously to degrade organic matter. Similar microbial communities could also be harnessed to ferment biomass to volatile fatty acids (VFAs) which are intermediate compounds formed during acidogenesis and acetogenesis by e.g., *Clostridium* and *Butyribacterium* [1]. VFAs are short chain carboxylic acids which can be harvested and further used as substrates and precursors in microbial or chemical transformations, e.g., in the production of polyhydroxyalkanoates (PHA) [2] or docosahexaenoic acid (DHA) [3]. In recent years, VFAs have become an anticipated resource- and cost-effective way to replace fossil resources in extensive industrial production of chemicals and fuels.

Compared to methane formed during anaerobic digestion, VFAs have significantly higher added value and more versatile utilization possibilities. For example, one ton of food waste treated with anaerobic digestion has been reported to be worth 76 €, while its value as unrefined caproic acid is €1000 (refined value 2000–3000 € per ton of food waste) [4]. However, there are still challenges in the production of targeted compounds from diverse biomasses, such as urban BWs. BWs, especially food waste, are composed of lipids, carbohydrates and proteins. Lipids are the less suitable for fast fermentation, but carbohydrates especially are easily converted into glucose which is immediately available for glycolysis and fermentation into VFAs [5]. With these types of biomasses, mixed culture fermentation should be favored because it can utilize various organic compounds and can be more tolerant towards changes in the substrate quality. Additionally, mixed cultures are not dependent on the aseptic culturing conditions required by single strain culturing [6].

Mixed culture fermentation could be operated in the same reactors as anaerobic digestion to lower the processing costs and even utilize the same inoculum during the process start-up. To optimize the VFA production, the growth of methanogens should be inhibited to enable formation and recovery of VFAs. There are several methods that can be used to make conditions unsuitable for methanogens, e.g., the use of 2-bromoethanesulfonate (BES) to block the enzyme functions of methanogens as well as thermal treatment/heat shock, pH increase/decrease or freeze-thaw treatment, to create unfavorable conditions for methanogens [1]. Extreme pH and temperature conditions inhibit the growth of methanogens, while favoring the viability of spore-forming bacteria from genera *Bacillus* and *Clostridium* [1,7]. The use of BES and pH alteration both require chemical additions, which can affect the economic and environmental footprint of the process, while the thermal and freeze-thaw treatments could be operated without chemicals but require energy inputs. Especially thermal treatment could be incorporated into the existing anaerobic digesters, as the digesters are often equipped with hygienisation/pasteurisation units. In continuous fermentation, the activity of methanogens could be reduced by the increase of the substrate loading rate, where the methanogenic activity is decreased due to VFA accumulation [1] and washing out of slow-growing archaea [8].

It is known that operation parameters such as pH, temperature and inoculum affect the VFA fermentation and the produced spectrum of acids through the optimization of different microbial pathways [1]. Thus, for a stable and profitable process operation, the microbial consortium within the fermenter should be optimized. However, there is currently very limited knowledge of the microbial communities and their functions [9], especially concerning the hydrolytic and fermentative bacteria [10]. During the last few decades, the advent and advance of next generation sequencing (NGS) technologies have had a major impact on the increase in knowledge of complex communities. With the increased understanding of the microbial communities and their changes during the mixed culture VFA fermentation, the maintaining of certain, defined microbial communities and modulation of metabolic pathways to produce desired acids as end-products could be enabled.

In this study, the aim was to screen different fermentation conditions for urban BW substrate using inoculum from an anaerobic digester. The microbial communities were analysed to determine the effects of the different treatments on the inocula and to study their dynamics during the fermentation process and VFA production. Optimal operation conditions were screened in two stages. In the first stage, VFA fermentation was tested in a 28-day batch experiment using thermal and freeze-thaw treated inocula, which were compared with the control (no treatment). In the second stage, a 10-day VFA fermentation experiment was set up with the best performing inoculum treatment (thermal treatment) to further look into the changes in microbial communities during the fermentation start-up and to target the days with the highest VFA production. 

## 2. Materials and Methods

### 2.1. Origin and Pretreatment of Materials

In this study, urban BW, i.e., a mixture of separately collected food and garden waste, was anaerobically digested to produce VFAs in two separate laboratory batch experiments. The BW sample was collected once and inoculum samples twice from a local waste treatment facility and from a full-scale biogas plant treating municipal and industrial biowastes (Envor Biotech Ltd., Forssa, Finland). The BW was macerated in a Retch Grindomix GM300 knife mill (Retch Gmbh, Germany) into a paste-like form. Prior to maceration, plastic bags and other harmful materials (e.g., bones) were sorted out. The pretreated BW was stored in a freezer (−20 °C) and thawed in a fridge (+4 °C) prior to use.

Both inocula samples were sieved (sieve size 1 × 1 mm) to remove coarse material before further treatment and batch experiments. In the first 28-day batch experiment, both thermal and freeze-thaw pre-treatments were applied to the inocula to prevent the growth of methanogens during batch experiments. In the second 10-day batch experiment, only thermal treatment was used. For the thermal treatment, the inocula were separately pre-treated by boiling at 94–100 °C for 30 min [11]. Each inoculum was boiled in 2–3 L portions, which were combined and stored in a fridge (+4 °C). For freeze-thaw pretreatment, inocula was frozen (−20 °C) in 500 g portions for 24 h after which they were thawed and stored in the fridge (+4 °C). Untreated inocula were stored in a fridge (+4 °C) and used as control inocula. Results from batch experiments with BW and control inoculum are referred to as CR, results with BW and thermally treated inoculum as TH and results BW and freeze-thaw treated inoculum as FR.

### 2.2. Batch Experiments

Untreated, thermally and freeze-thaw treated inocula were used in 28-day (first experiment) and 10-day (second experiment) batch BMP (biochemical methane potential) tests. The assays were performed in mesophilic (37 °C) conditions using automated testing equipment (Bioprocess Control Ltd., Sweden) as previously in Tampio et al. [11]. Substrate and inoculum were added to the bottles according to the VS_substrate_ to VS_inoculum_ ratio of 0.5 [11,12,13] and distilled water was added to achieve total liquid capacity of 400 g. From the formed biogas, CO_2_ was fixed with a 3 M sodium hydroxide solution and the volume of methane gas was determined by water displacement. It should be noted that the gas measured after the CO_2_ fixation can also contain other components, such as H_2_. The VFA content and microbial communities during the experiment were analyzed from the BMP bottles using destructive sampling by terminating triplicate test bottles at a time from each treatment. During the test, triplicate bottles were terminated after 7, 14, 21 and 28 days in the first experiment, and after 1, 3, 6 and 10 days in the second experiment.

### 2.3. Chemical Analyses and Calculations

BW and inocula were tested for their chemical composition prior to batch experiments and after terminating each batch bottle. The pH was determined using a VWR pH100 pH-analyzer (VWR International). The total and volatile solids (TS and VS) were analyzed according to SFS 3008 [14]. For the analysis of the soluble chemical oxygen demand (SCOD), samples were diluted 1:10 with distilled water and centrifuged as described in [15] and analyzed according to SFS 5504 [16]. VFAs (acetic, propionic, iso-butyric, *n*-butyric, iso-valeric, valeric and caproic acids) were analyzed using an HP 6890 gas chromatograph (Hewlett-Packard, Little Falls, USA) with a 10 m × 0.53 mm × 1 µm HP-FFAP capillary column (Agilent Technologies, USA) and a flame ionization detector as described in more detail in [15].

Methane yields in the BMP assays were converted to STP conditions (0 °C, 100 kPa) according to the ideal gas law. The methane production of the inoculum was subtracted from the results containing both substrate and inoculum to determine the methane production of the substrate. In the case of pretreated inoculum, the gas production of the pretreated inoculum was used. Methane yields in the batch assays were calculated by dividing the cumulative methane production by the VS of the added substrate. The theoretical COD equivalences 1.066, 1.512, 1.816, 2.036, and 2.204 gCOD/L were used for acetic acid, propionic acid, butyric acid, valeric acid, and caproic acid, respectively [17].

### 2.4. DNA Extraction, Sequencing of the Region V3-V4 of 16S rDNA Gene

To study the differences on microbial communities during the process, duplicate (on the 28-day experiment days 7, 14 and 21, on the 10-day experiment days 1, 3 and 6) or triplicate (last day of sampling, day 28 or day 10) samples from both experiments were collected for DNA extraction. DNA was extracted from 0.25 g wet weight using FastPrep DNA extraction kit for soil (MP Biomedicals, LLC, Solon, OH, USA) according to manufacturer’s instructions, as used previously [18]. DNA concentrations were measured with Nano Drop and they were adjusted to 20 ng/µL. Extracted DNA was used for the Illumina amplicon library preparation at the Finnish Functional Genomics Centre (FFGC, Turku, Finland). DNA was amplified using the 341F/805R set of primers for bacteria and 349F/806R set for archaea targeting the V3V4 region. Library preparation was done according to Illumina’s Metagenomic Sequencing Library Preparation protocol (part # 15044223 Rev. B). Sequencing was conducted using an Illumina MiSeq platform (FFGC, Turku, Finland).

### 2.5. Sequences and Statistical Analyses

Sequences were processed using the Mothur 1.42.3 software package [19]. Bioinformatics processing of the Illumina reads followed the Mothur Miseq Standard Operating Procedure (SOP) [20]. Sequences were rejected if they (a) did not match the primers (2 differences allowed), (b) contained ambiguous nucleotides, (c) were less than 400 bp in length, (d) had an average quality score below 30, or (d) had homopolymer sequences longer than 8 bp. Sequences were aligned against the Greengenes 13_8 database ([21,22] http://greengenes.secondgenome.com) as reference alignment using kmer = 8 method, chimera sequences were checked and removed using UCHIME [19]. Reads were then clustered into operational taxonomic units (OTUs) using 97% sequence similarity thresholds. OTUs present in a lower relative abundance than 0.01% of total reads were removed. The samples were rarified to 9461 and 1019 sequences for bacteria and archaea, respectively. The sequencing data obtained in this study is submitted to the NCBI Sequence Read Archive under the study accession number PRJNA623485.

Good’s coverage, rarefaction curve analyses and alpha diversities including community diversity (Shannon index) and richness (Chao1) were calculated using Mothur. Kruskal–Wallis test was used to measure significant differences on the alpha diversity indexes. Meanwhile, Mann–Whitney pairwise test was used to test the differences (Shannon index and Chao1) between each treatment. The microbial community structure was explored by non-metric multidimensional scaling plot (NMDS) using Bray–Curtis dissimilarity index on the Hellinger transformed data values of OTUs, and the statistical comparison between treatments was determined through a permutational analysis of variance (PERMANOVA, 999 permutations) using the Bray–Curtis index. Unweighted pair-group method with arithmetic mean (UPGMA) phylogenetic tree was constructed based on weighted UniFrac distance. Statistical analyses were done with the normalized, centered and scaled relative abundance of taxonomically identified microbial communities at different levels using the vegan package in R [23], and the program PAST 3.0 [24]. Spearman rank correlation was conducted to identify the relationship between relative abundance of archaea and bacteria at different levels (the first two principal components) and operational conditions (acetic, propionic, iso-butyric, *n*-butyric, iso-valeric, valeric and caproic acids), using PAST 3.0. The significances were calculated using permutation test.

## 3. Results

### 3.1. BW and Inocula Characteristics

BW collected from a local waste management facility was used for both 28-day and 10-day experiments. Between experiments, BW was stored in a freezer, which slightly affected the BW characteristics, by solubilizing organic matter (SCOD increase from 91 to 120 g/L, Table 1). The inoculum for 28- and 10-day experiments was collected at different time points. The thermal treatment affected the inoculum by increasing TS and VS 26%–44% as water was evaporated. Simultaneously organic matter was solubilized, which increased inoculum SCOD by 145%–155%. The freeze-thaw treatment had a similar but weaker effect on the inoculum characteristics, as the TS and SCOD increase were 13% and 16%, respectively.

### 3.2. VFA Fermentation

**Figure 1:**
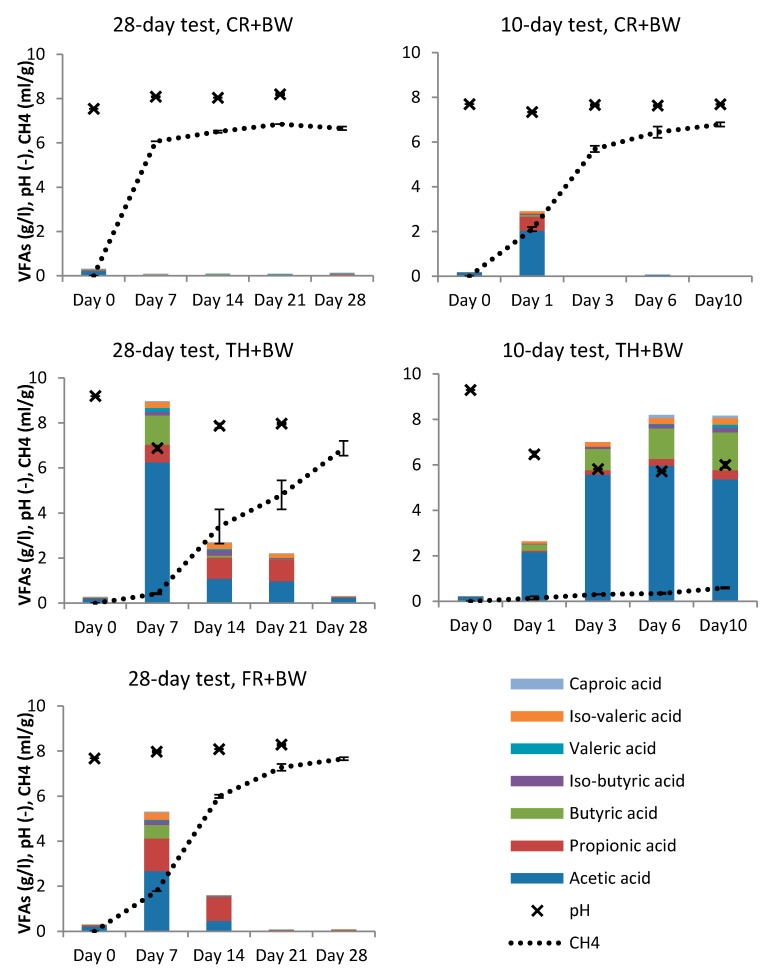
Volatile fatty acids (VFAs) concentration (g/L), pH and methane production (mL/g bottle liquid content) in test bottles during 28 and 10-day experiments. Results are averages and standard deviations from triplicate bottles. In 28-day experiment day 7 CH_4_ results *n* = 1 for CR and TH, *n* = 2 for FR. Test bottles with biowaste (BW) and control inoculum (CR), BW and thermally treated inoculum (TH) and BW and freeze-thaw treated inoculum (FR).

During the 28-day experiment, VFA production from BW was detected with both thermal and freeze-thaw treated inocula (Figure 1, Table S1.1 in the Supplementary Material S1). The highest VFA production was observed on the first sampling date, on day 7 (total VFAs 9.0 g/L in TH and 5.1 g/L in FR). After day 7, VFA concentrations decreased and were ceased by day 21 in FR and by day 28 in TH. Methane production was observed to increase as VFA production decreased when using thermally or freeze-thaw treated inocula. With control inoculum, no VFAs were detected (<0.1 g/L) during the 28-day experiment, while methane production was rapidly increased indicating that methanogens, and also acetogens, present in the inoculum consumed majority of the produced VFAs. On day 28, the maximum methane production was 430 m^3^/tVS for BW with control inoculum (see Table S1.2 in Supplementary Material S1), while inoculum treatment increased methane production from BW with both freeze-thaw (462 m^3^/tVS) and thermal treatment (563 m^3^/tVS), respectively.

In the 10-day experiment, the aim was to analyse in more detail the VFA production during the start-up phase of the digestion process. The shorter experiment was done only with thermally treated inoculum, as it showed higher VFA production and lower methane production compared to the FR treatment in the 28-day experiment. The first days of the digestion process seemed to be the most crucial. The pH levels decreased from the initial 9.3 to 6.5 during the first day of digestion with VFA production of 2.6 g/L (Figure 1). The highest VFA production was detected on day 6 with a total VFA concentration of 8.2 g/L. Methane production was very low during the 10-day experiment with TH (maximum methane production 48 m^3^/tVS on day 10) compared to CR (435 m^3^/tVS). In CR samples, VFA concentration of 2.9 g/L was observed on day 1, after which the VFA production was negligible as methane was produced.

In fermentations using treated inocula, acetic acid was the main acid produced in the start-up phase (until day 10) accounting for 50–80% of the total VFAs produced (Figure 1, see also Table S1.3 in Supplementary material S1). After day 10, the share of propionic acid was observed to increase to >30% in both TH and FR. However, in the short 10-day experiment, thermal treatment was observed to favor butyric acid production (10–20% of VFAs on days 1–10), while the production of propionic acid increased after day 10 (35–45% on days 14–21). Freeze-thaw treatment favored propionic acid production (27–67%) and also butyric acid production to some extent (1–10%).

### 3.3. Microbial Community Analyses Based on 16S rRNA Gene Amplicon Sequencing

#### 3.3.1. Alpha Diversity in the BMP

Overall 832,427 bacterial and 530,636 archaeal nonchimeric, good-quality sequences were retrieved for all the samples (45 samples), representing 668 bacterial and 118 archaeal operational taxonomic units (OTU) grouped at 97% of similarity for the three tested conditions (CR, TH and FR). Rarefaction curves showed that the bacterial and archaeal communities were sampled sufficiently (Figure S1.1 in the Supplementary Material S1). Thermal treatment of the inoculum decreased substantially the number of retrieved sequences. The averages of sequences per sample were 18,499 and 11,792 for bacteria and archaea. Due to the lower number of sequences in TH samples, and in order to avoid losing information, the samples were rarified to 9458 and 1019 for bacteria and archaea, respectively. Good’s coverage of most of the samples was over 95% indicating that the 16S rDNA sequences identified in these samples represent the majority of bacteria present in the samples of this study. Lower coverages were obtained for three TH samples.

**Figure 2:**
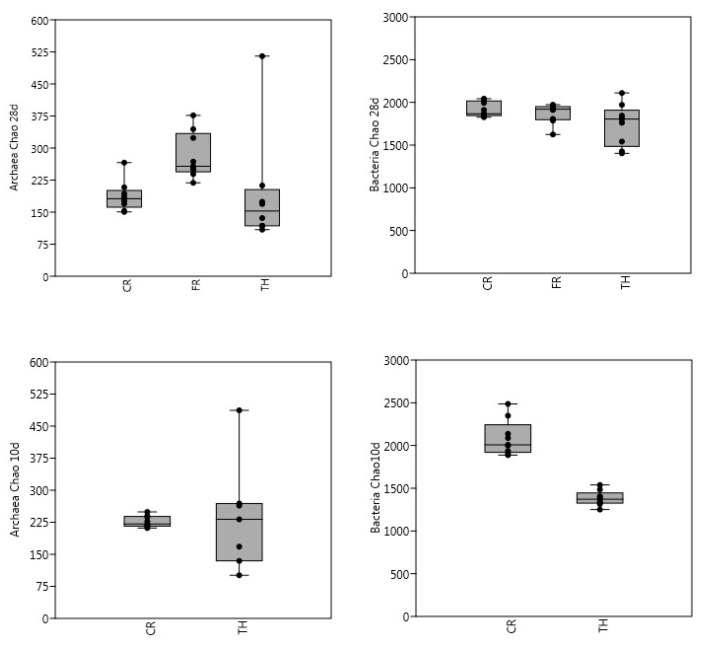
Archaeal and bacterial richness (Chao1) as measured from amplicon sequence data in batch bottles with BW and control (CR), freeze-thaw (FR) and thermally (TH) treated inocula. Length of the experiment was 28 days (treatments CR, FR and TH, figures above) and 10 days (treatments CR and TH, figures below). For each sample, the 25–75 percent quartiles are drawn using a box. The median is shown with a horizontal line inside the box. The minimal and maximal values are shown with short horizontal lines ("whiskers"). The dots are the sample values included.

**Figure 3:**
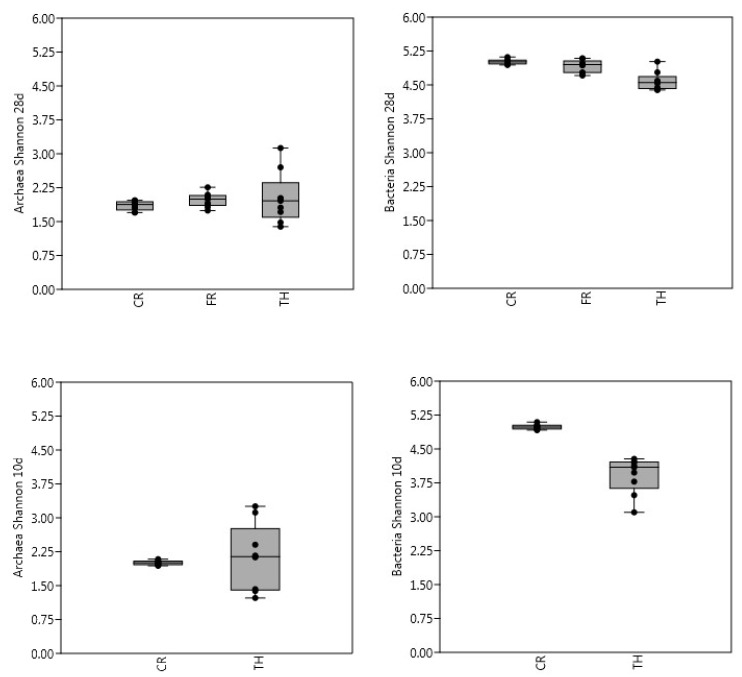
Archaeal and bacterial Shannon diversity index in batch bottles with BW and control (CR), freeze-thaw (FR) and thermally (TH) treated inocula. Length of the experiment was 28 days (treatments CR, FR and TH, figures above) and 10 days (treatments CR and TH, figures below). For each sample, the 25–75 percent quartiles are drawn using a box. The median is shown with a horizontal line inside the box. The minimal and maximal values are shown with short horizontal lines ("whiskers"). The dots are the sample values included.

In general, for archaea there were no significant differences in richness between samples when using Kruskal–Wallis test, but when using the Mann–Whitney pairwise test, significant differences on the 28-day experiment between FR with CR and TH were observed. For bacteria, there were significant differences on both experiments (28-day and 10-day) using Kruskal-Wallis. However, when analyzing pairwise significances, only CR was significantly different to TH (Figure 2). Compared with the bacterial community, the Shannon index was generally lower for the archaeal community. There were no significant differences on the Shannon index of archaeal communities between experiments or between the pairwise samples. TH showed lower bacteria diversity index averages than CR and FR, indicating lower microbial richness and evenness. TH was significantly different from CR on both 28-day and 10-day experiments when using Kruskal-Wallis. From day 1 onwards, extensive changes in microbial diversity were detected in TH samples, mainly due to the influence of microbial community present in the BW substrate. When the treatments were compared, the overall diversity was evidenced to be highest in the CR samples (Figure 3). The highest bacterial diversity and archaeal species richness were found in the CR samples, followed by FR. The lowest diversity and species richness for bacteria were detected in the TH samples, where the inoculum had been thermally treated. Diversity and species richness were higher for bacteria than for archaea. The number of bacterial OTU-97% was eight times bigger than that for Archaea as reported previously by [9].

**Figure 4:**
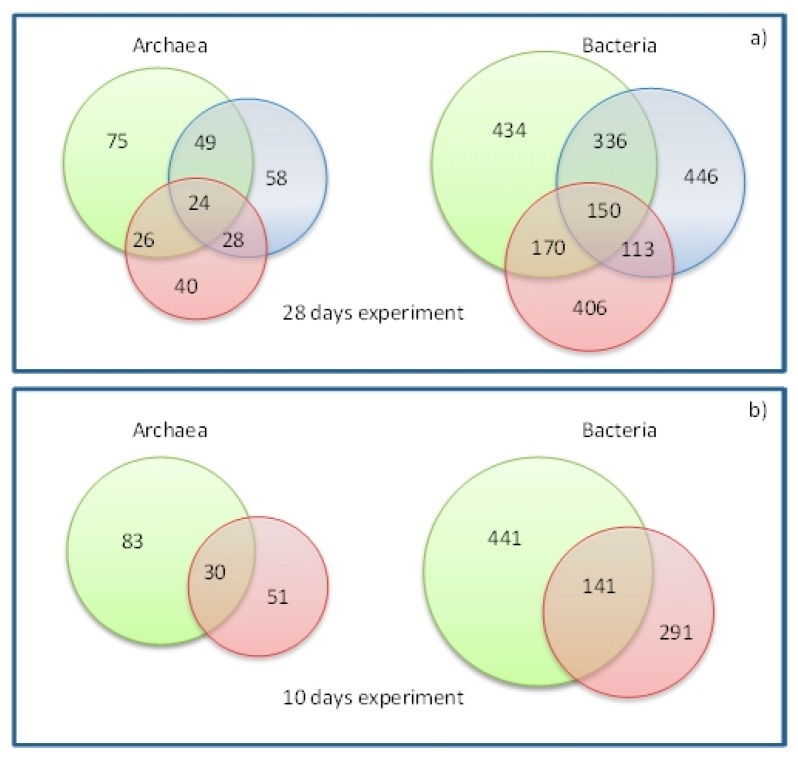
Venn diagrams representing the shared OTUs between samples from the batch tests after the different inoculum treatments, (**a**) 28-day experiment (days 7, 14, 21 and 28), and (**b**) 10-day experiment (days 1, 3, 6 and 10). Each set of samples is differentiated by the colors. Green color refers to the CR samples with BW and control inoculum, red color to TH samples with BW and inoculum thermal treatment, and blue color to FR samples with BW and inoculum freeze-thaw treatment.

Venn diagrams (Figure 4) show the number of shared and total OTUs regardless of their abundances, which were compared between the 28-day experiment (with CR, FR and TH) and the 10-day experiment (CR and TH). In the 28-day experiment, the total amount of common OTUs was 23.81% and 20.80% for archaea and bacteria respectively, and in the 10-day experiment, 20.86% and 19.69% for archaea and bacteria, respectively. The Venn diagram analysis implied that the archaeal communities of the three batch fermentations were diverse.

#### 3.3.2. Beta Diversity

**Figure 5:**
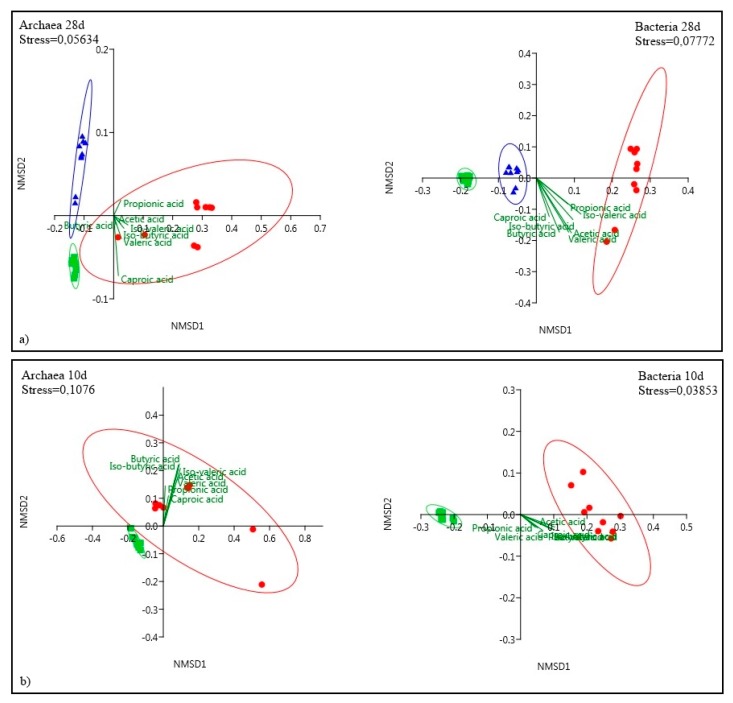
NMDS plots of the (**a**) 28-day experiment and (**b**) 10-day experiment. Bray–Curtis dissimilarity index on Hellinger transformation of the amounts of OTUs has been applied. The ellipses indicate the probability of finding the samples with a 95% interval of confidence. Individual samples are represented by symbols, colored based on the inoculum treatment: control (square green), freeze-thaw (triangle blue) and thermal (dot red). Shepherds stress is indicated on the plots.

Beta diversity or grouping of samples based on the inoculum treatments and sampling days was evaluated by using Bray–Curtis as the dissimilarity measure and nonmetric multidimensional scaling (NMDS) as the ordination method (considering the presence/absence of OTUs as well as the relative abundance of OTUs). Distance-based permutational multivariate analysis of variance (PERMANOVA) was used to assess significant differences with respect to treatment. Significance was defined at *p <* 0.05 after 999 permutations as implemented in PAST 3.0. The structure of microbial communities in the batch experiments, assessed by two-dimensional NMDS plots based on Bray–Curtis dissimilarities, showed marked differences between inoculum treatments in both archaea and bacteria (PERMANOVA analysis for treatments *p <* 0.05). Association between TH treatment and VFA production was observed (Figure 5).

The relationships between the community structures revealed by NMDS were further tested by comparing the between-group weighted Unifrac distances and unweighted pair-group method with arithmetic mean (UPGMA) tree using the Bray–Curtis index with 999 bootstrap (for bacterial and archaeal communities independently, Figure S1.2 in the Supplementary Material S1). Consistent with the NMDS plot, the between-group distances of TH and CR, as well as TH and FR, were significantly higher than that of FR and CR. This data suggested that the microbial community structures between the TH and other treatments were significantly different. The overall similarity was higher for the FR and CR, than to TH. Communities differed clearly between day 1 and the other sampling days of the TH, similarity values evidenced that the differences were stronger in the beginning of the period (Figure S1.1 in the Supplementary Material S1).

#### 3.3.3. Phylogenetic Analyses

The relative abundance of data from the sequences retrieved from the all the batches (all inoculum treatments and both experiment lengths), was used in a two-way PERMANOVA with a Bray–Curtis index to test the effect of treatments and interaction of treatment and sampling day. Significant differences between treatments are indicated on Supplementary Material S1 (Table S1.4, *p* < 0.05). The relative abundance of the microbial communities on the CR samples were stable during both the 10-day and 28-day experiments, while FR and TH showed clear changes in the compositions during the sampling period.

Archaeal community analyses showed class Methanomicrobia abundances of 68.33 ± 2.17% in the 10-day and 68.50 ± 2.88% in the 28-day experiment in CR, while in the TH the abundances were 43.74 ± 4.93% and 70.34 ± 12.94%, having a clear tendency to increase over time. The second most abundant class of archaea was Methanobacteria, abundances in CR were 16.57 ± 2.38% on the 10-day experiment and 15.91 ± 1.41% on the 28-day experiment. In TH, Methanobacteria had a clear tendency to diminish during the experiment as abundances of 38.93 ± 5.24% were found on the 10-day and 17.47 ± 9.83% on the 28-day experiment. The composition of the inoculum was not significantly different in CR between the 10- and 28-day experiments, thus it was concluded that the effects on the communities were modulated by the treatment and the sampling day (or days of incubation).

**Figure 6:**
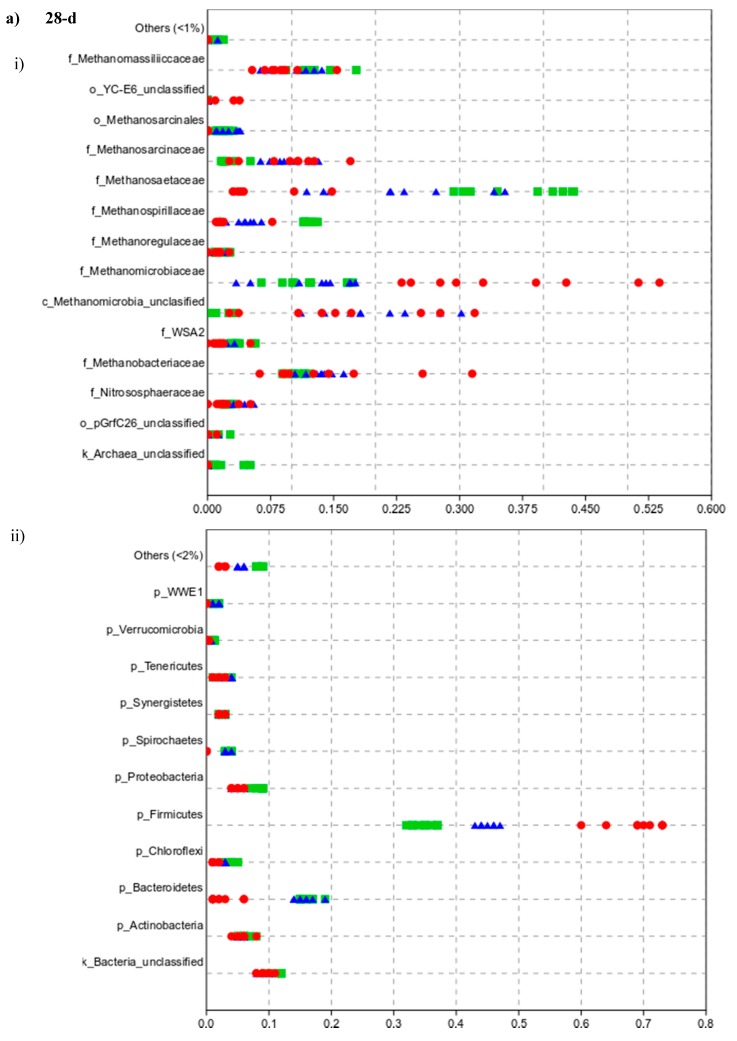

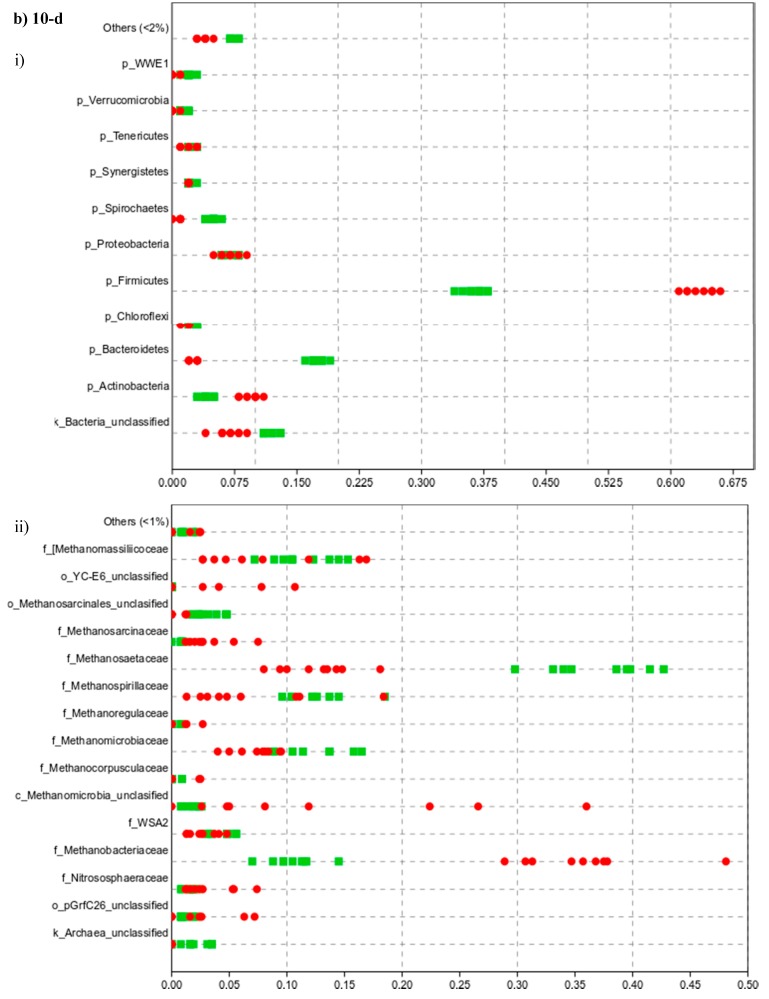
Jitter plot of the relative abundances of archaea at the family level (i) and bacteria at the phylum level (ii). (**a**) 28-day (above) and (**b**) 10-day (below) batch experiments. Green squares refer to the CR samples with BW and control inoculum, red dots to TH samples with BW and inoculum thermal treatment, and blue triangles to FR samples with BW and inoculum freeze-thaw treatment (for average values and statistical significance see Supplementary Material Table S1.4).

Composition at the family level remained stable for the CR but in TH there was a shift in the community over time. *Methanosaetaceae* was more abundant in samples with no inoculum treatment (% of abundance±SD as 37.08 ± 4.34% and 37.24 ± 5.87% for 10-day and 28-day experiment in CR, respectively, and, 12.58 ± 3.12 for 10-day and 5.62 ± 4.11% for 28-day experiment in TH). *Methanobacteriaceae* dominated the archaeal community when the inoculum was thermally treated (35.72 ± 5.64% for the 10-day and 15.06 ± 8.46% for the 28-day experiment with TH) with a clear decrease, while in CR this family was stable (10.43 ± 1.13 and 10.52 ± 2.11% for 10- and 28-day experiments). *Methanomicrobiaceae* accounted for 12.30 ± 3.84% and 12.7 ± 2.69% on the 10- and 28-day experiments with CR and 7.31 ± 1.91% and 36.03 ± 11.35% in TH, with a tendency to increase during the time. Significant differences between the CR and TH for the above mentioned families were detected. The distribution of these families can be observed on the jitter plot (Figure 6, the values and significances are presented in the Supplementary Material S1 Table S1.4). The appearance of *Methanobacteriaceae* was shown to correlate positively especially with the presence of acetic acid and butyric acid.

The jitter plot (Figure 6) clearly shows that the most abundant bacterial phyla among all samples with different inoculum treatments were Firmicutes. In samples with the thermally treated inoculum, Firmicutes was the most abundant phylum (63.67 ± 1.73% and 69.11 ± 4.46% for 10 and 28-day experiments, followed by Actinobacteria (5.78 ± 1.09% and 9.44 ± 1.00%), Proteobacteria (6.89 ± 1.17% and 5.00 ± 0.87%) and Bacteroidetes (2.56 ± 0.53% and 2.56 ± 2.07%). In the CR, Firmicutes was also the most abundant (36.44 ± 1.33% and 34.67 ± 1.80), followed by Bacteroidetes (17.44 ± 0.88% and 16.11 ± 1.36%), Proteobacteria (7.11 ± 0.93% and 8.56 ± 0.73%, not significantly different between 10 and 28-day experiments), and Actinobacteria (4.11 ± 0.78% and 6.00 ± 1.00%). The percent abundances were dependent on the inoculum treatment. Firmicutes were dominated by phylotypes belonging to orders Clostridiales and Lactobacillales. When analyzing the bacterial communities to the order level, it could be observed that Clostridiales was the most abundant in TH (45–60%), followed by FR (30–37%) and CR (20–23%).

**Figure 7:**
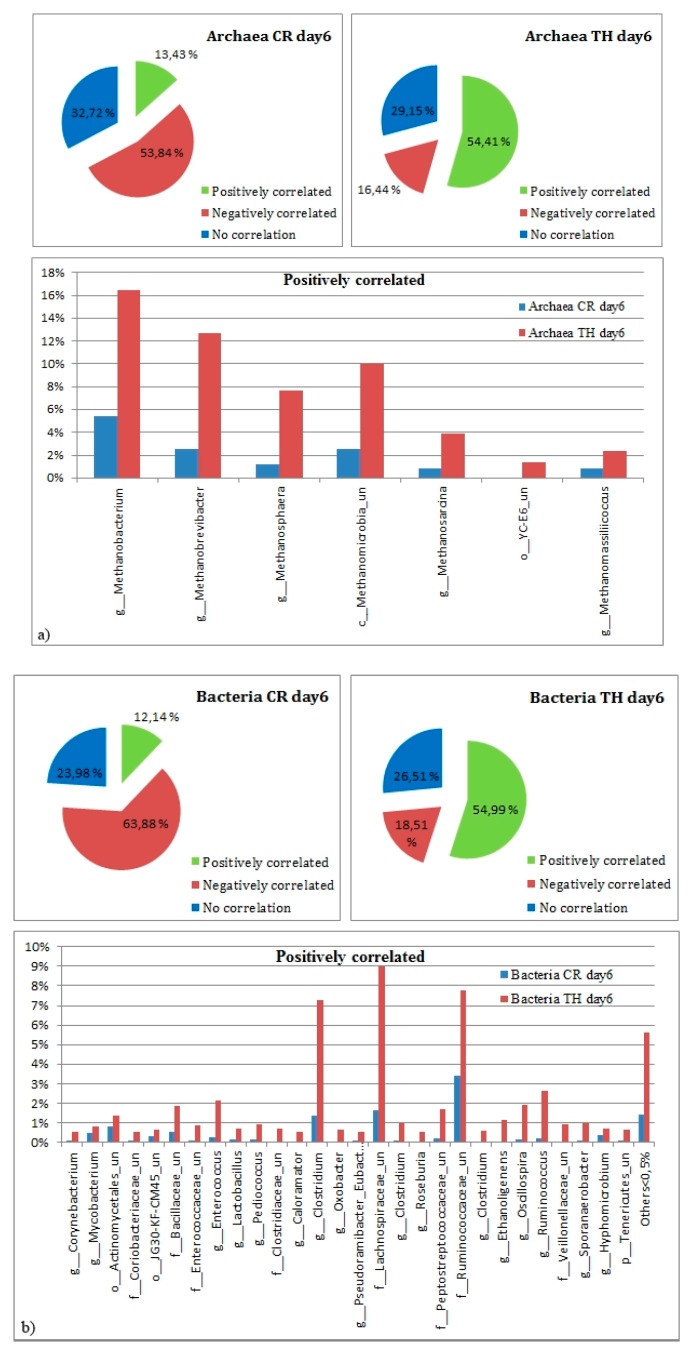
Relative abundances with detailed information on the classified OTUs positively correlated to the VFA production on day 6. (**a**) Archaea, (**b**) bacteria. Day 6 was chosen to be the one where highest VFA amounts were produced. Unclassified (un). For detailed abundance values and significance see the Supplementary Material S2.

It was corroborated in the first 28-day experiment that the highest amount of VFAs was produced during the first 10 days of the fermentation process, and that the thermal treatment was the most successful inoculum treatment for increased VFA production and inactivation of methanogens. Therefore, in a shorter 10-day experiment the focus was on the thermal treatment, which was compared with control inoculum to further study the relationships between microbial communities and VFAs production. From the 10-day experiment, the sequences were analyzed at the family taxonomic level. The sequences correlating positively to the production of VFAs were linked to the families *Clostridiaceae* (8.19–14.17% in TH samples and 1.56–2.52% in CR), *Lachnospiraceae* (2.53–12.25% in TH and 1.57–6.16% in CR), *Ruminococcaceae* (0.84% on day 1 in TH to 15.65% on day 10, and 4.6 ± 0.34% for days 1–10 in CR), *Enterococcaceae* (1.06–7.82% in TH and 0.5–0.66% in CR) and *Lactobacillaceae* (1.09–10.50% in TH and 0.16–1.58% in CR). The samples were analyzed to the genus level in both archaea and bacteria, finding a core a microbial community associated to VFA production on different proportions on CR and TH (Figure 7). We focused on the positive correlations established between microbes and VFA, and whether those genera were significantly different across the treatments. The synergistic relationship established between those microorganisms related to VFA production were also explored (see Supplementary Material S2).

**Figure 8:**
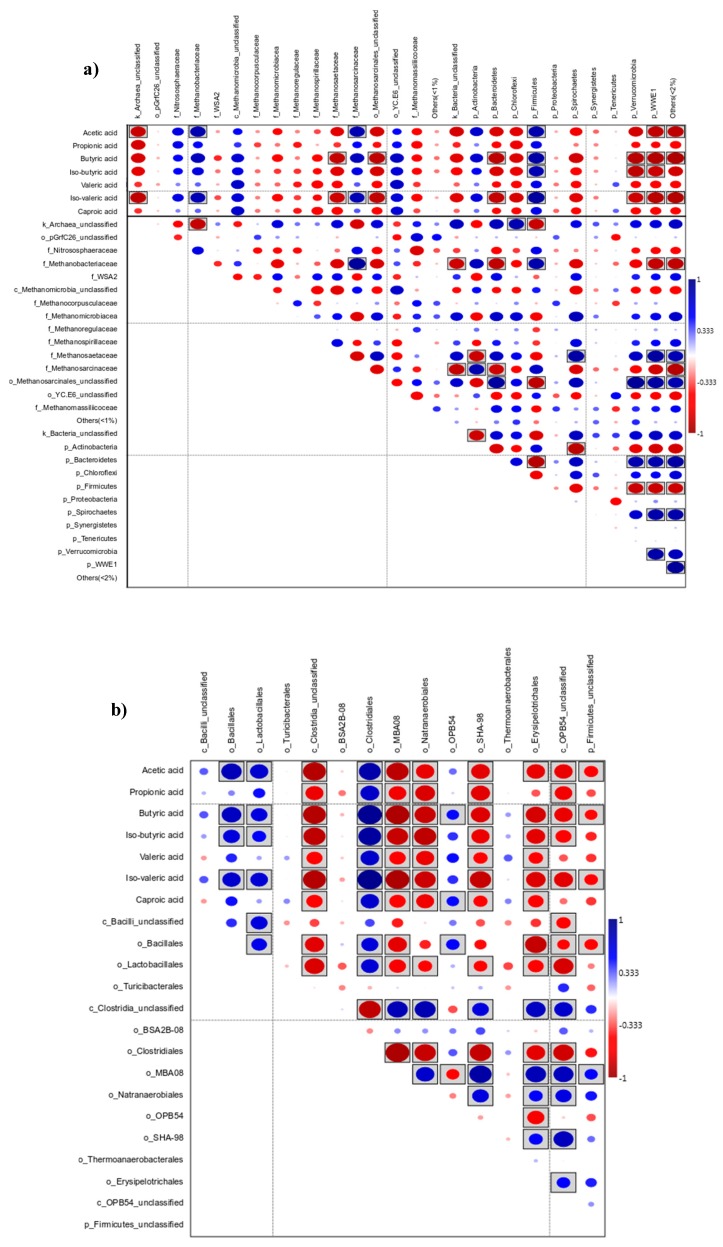
Spearman rank correlation between the microbial composition and the produced VFAs for the 10-day experiment, (**a**) Bacteria and archaea at family and phylum level; (**b**) bacteria at the order level (on the phylum Firmicutes), significance (*p* < 0.05) labeled as boxed ellipses.

The Spearman rank correlation between the microbial community and the produced VFAs were analyzed for the 10-day treatment (Figure 8a). A positive correlation was found especially between production of VFAs and Firmicutes. This phylum appeared in higher numbers in TH, and therefore, within Firmicutes, the microbial community was studied to a deeper level (Figure 8b). Correlation was calculated both for the archaeal and bacterial communities at the genus level (only positive correlations to VFA ≥ 0.5 with *p* < 0.005 are shown, see the Supplementary Material S2 for detailed data). The microbiome core in different treatments in the 10-day experiment was also defined at the genus level for archaea and bacteria on the highest VFA production day (day 6, Figure 7).

## 4. Discussion

### 4.1. Effect of Inoculum Treatment on the Fermentation

The thermal and freeze-thaw inocula treatments were observed to partially inactivate some of the archaea and bacteria, although both inocula treatments increased VFA production. During the 28-day experiment, the highest VFA concentrations were observed on the first day of sampling (9 g/L, day 7) after which the concentrations were decreased to 2–3 g/L by the next sampling on day 14. Thus, it was evident that with inoculum treatments, only a partial deactivation of methanogens was achieved as the methane production was recovered after some time. In FR samples, no pH decrease was observed and the VFA production almost ceased on day 21, while methane production was achieving a plateau. Previously, the partial deactivation of methanogens after freeze-thaw treatment and the increase in methane production has been also reported by Phalakornkule et al. [7].

With TH treatment, the deactivation was more effective and lasted longer as VFAs were still produced on days 21 and 28, while methane production was still increasing. Fewer OTUs in TH than CR or FR treatment indicated that thermal treatment effectively reduced both bacterial and archaeal diversity. As shown on the NMDS plots, significant differences in the microbial communities were found in the different treatments. The microbial community of TH samples was linked to improved VFA yields, especially on day 6 of the 10-day experiment.

The heat treatment had an impact on the inoculum. Bacteria which are not able to sporulate are known to be killed during this kind of treatment, allowing the spore-forming bacteria to become the most relatively abundant after a couple of days of fermentation. However, it is known that methanogens can survive heat treatment if not sufficient treatment time or temperature is applied [25]. Previously, a lower treatment temperature of 80 °C for 15 to 30 min has been reported to be the most effective concerning the VFA production from glucose using anaerobic digester inoculum [26]. In previous studies, where archaea were subjected to heat drying at 104 °C for 12 h, it was proven that the archaea were not completely inactivated, being increasingly resistant in the order *Methanospirillum* > *Methanosaeta*> *Methanoculleus* [27]. In the present study we found also other “resistant” archaea, *Methanosaeta* (18%), which seemed to be the most heat resistant archaea followed by *Methanobrevibacter* (16%), g_vadinCA11 (16%), *Methanobacterium* (8%), *Methanosphaera* (7%), *Methanoculleus* (6%), and *Methanospirillum* (4%). All of these were present at day 1 in TH. When considering the archaeal community, as described previously [28,29], the most abundant family associated with methane production was *Methanosaetaceae*, which was found in CR with a relative abundance of 40%.

Because of the thermal treatment, bacteria, like clostridia, which are able to sporulate, can increase quickly in numbers after reaching suitable growth conditions. Lactobacillales are not heat resistant bacteria, but due to the addition of the BW as a feed material, it was assumed that the communities were enriched, as the BW source contained up to 40% of Lactobacillales. In the fermentation with TH inoculum, Lactobacillales were detected in the beginning of the acidogenic fermentation (day 1 at 20% of abundance after which the amounts were reduced to 4% on day 10).

### 4.2. Connections between VFAs and Microbial Communities

Around 50% of the VS in food wastes consist of carbohydrates, while both proteins and fats contribute to 10–30% of the organic matter. Despite a high theoretical conversion efficiency of glucose (assuming it as the most abundant carbohydrate component) into acetic acid, this acid comprised only 40–80% of the produced VFAs in all tested samples, depending on the sampling day. The retention time was observed to have an effect on the spectrum of VFAs as reported previously by [30]. A mix of acetic acid (60–80%) and butyric acid (10–20%) was observed during the first 10 days of the fermentation with TH treated inoculum. During days 14–28 the acid spectrum consisted mainly of acetic acid (40–70%) and propionic acid (10–45%). Furthermore, the inoculum treatment affected acid formation, where acetic (30–50%) and propionic acid (30–70%) were in abundance in FR, but some butyric acid (10%) was also produced. According to this, it seemed that the inoculum treatment also had some effect on the VFA spectrum. Previously, it has been suggested that the inoculum is responsible of the produced VFA spectrum [1], although the inoculum type does not affect VFA production efficiency [31]. It seems that the treatment of inocula in different conditions (thermal and freeze-thaw) enriched different microbial communities, which further changed the metabolic pathways during BW degradation and resulted in different VFA spectra, which has been also reported previously [8].

In this study, seven archaeal phylotypes with the highest correlation for VFA production were shown to correspond to 42–72% of the total archaeal community in TH, while in CR they represented 13% to 17 % of the defined communities (see Supplementary Material S2). Similarly, abundance of bacterial communities positively correlating with VFA production was higher in TH, accounting for 48–55% in TH and 12–19% in CR. We found a correlation between *Methanobacteriaceae* and VFA production, suggesting that a symbiosis was established between this family and some Firmicutes. As other studies have suggested, Methanobacterium (belonging to the *Methanobacteriaceae* family) seemed not to be affected by VFA concentration. This is a genus containing hydrogenotrophic methanogens, and it has been observed that they are syntrophic partners of acetate oxidizers [32].

The averages of relative abundance of bacteria of phyla Firmicutes, Actinobacteria, Bacteroidetes and Proteobacteria were 63.67%, 9.44%, 2.56% and 6.89% respectively for the TH samples. Previously, Atasoy et al. [31] reported the most abundant phyla involved in the fermentation of glucose to be Firmicutes, Bacteroidetes, Proteobacteria and Chloroflexi, of which especially Firmicutes (genus *Clostridium*) were connected to the highest VFA production. In the present study, the results showed that the thermal treatment of the inoculum was favorable to the growth of bacteria belonging to the phylum Firmicutes but repressed the growth in phyla of Bacteroidetes and Proteobacteria. Bacteria in the phylum Firmicutes are known to be related to the conversion of simple sugar into organic acids. Bacteria of classes Clostridia (phylum Firmicutes) have been found to degrade organic matter to produce various organic acids [33].

Clostridiales and Lactobacillales were present in all the samples but were found to be more abundant during the VFA fermentation with thermally treated inoculum. These orders have been widely associated with VFA production, and mixtures of acetic, *n*-butyric, caproic and lactic acids have been shown to be characteristic for clostridial fermentation [10]. The Clostridiales order is known to have bacteria with higher hydrolytic and fermentative activity, which could be related to VFA formation. Clostridiales have been shown to contribute especially to butyrate production [34], while Lactobacillales have been shown to positively associate with acetate, butyrate and propionate [35]. The Lactobacillales order contains several genera, and they are known to produce lactic acid as the major fermentation product from sugars and can utilize easily fermentable short chain sugars for growth [36,37]. Members of this phylotype could produce high concentrations of both lactic and acetic acids during the first 2–3 days of the acidogenic fermentation [10,36]. Lactobacillales have a high acid tolerance, surviving pH values of 5 and lower, besides they can acidify the media, which was detected on day 3 as the pH of the TH sample was 5.8, after that a shift on bacterial community is observed. This phylotype remained present in minor proportions during the entire experiment despite being overgrown from other bacteria mainly belonging to family *Clostridiaceae*. This might be explained by the fact that the composition was analyzed based on DNA, which does not necessarily reflect the actual activity of the organisms. After 2–3 days of the fermentation with thermally treated inoculum, the metabolic performance of the system changed and the Lactobacillales order was gradually replaced by phylotypes affiliated to the order Clostridiales.

Previous studies have pointed out the strong hydrolytic activity of Clostridiales [38]. They can effectively ferment sugars, and therefore they could grow on the BW, which may contain 30%–60% of carbohydrates. Mainly three families belonging to this order contributed to the overall highest relative abundances; *Clostridiaceae*, *Ruminococcaceae* and *Lachnospiraceae*. The *Ruminococcaceae* and *Lachnospiraceae* belong to the order Clostridiales. *Ruminococcaceae* can hydrolyze a variety of polysaccharides by different mechanisms, e.g., producing cellulolytic enzymes [39]. They are also known to produce short-chain fatty acids, VFAs [40]. Moreover, they are able to ferment hexoses as well as pentoses. Various genera of *Lachnospiraceae* are known to produce large amounts of *n*-butyric acid, acetic acid and carbon dioxide through the fermentation of carbohydrates [41]. In the present study, *Ruminococcaceae* and *Lachnospiraceae* prevailed, and were accompanied by the formation of caproic and acetic acids.

There are already well-known associations between hydrogen-producing bacteria, e.g., *Ruminococcaceae*, and hydrotrophic methanogens *Methanomicrobiaceae* [42]. Furthermore, as described previously, when *Ruminococcus* and *Methanobacterium* were grown together, acetate was the major fermentation products, while H_2_ did not accumulate; and large amounts of CH_4_ were formed [43]. In our experiment, we observed at the end of the 10-day experiment how the abundances of those two genera increased along with the CH_4_ production. Since most of the species and their ecological functions in biogas processes are still unknown, analyses of microbial compositions under various conditions are still required. These data would provide statistical support for the findings and understanding of how microbes contribute to the process and their syntrophic relation. Moreover, taxonomic classification conducted by similarity searches against 16S rRNA gene reference databases means that the classification depends on the completeness and correctness of the database. Frequent updates of the databases are definitely needed since a high proportion of the obtained sequences can only be classified on higher taxonomic ranks.

## 5. Conclusions

The results of our study revealed important differences on the microbial communities after the inoculum treatment compared to the control. Thermal treatment of the inoculum was shown to be the most effective treatment although it was able to inactivate methanogens only partially, and the highest VFA production occurred at day 6 of the batch fermentation. The high relative abundances of hydrolytic bacteria such as *Clostridiaceae*, *Ruminococcaceae* and *Lachnospiraceae* were strongly correlated to VFA production, and associated to the presence of *Methanobacterium*. Microbes with low abundances may also correlate with important biochemical parameters and have substantial effects on the process performance. During the fermentation experiments, the microbial communities started to degrade VFA to produce methane gas after day 6, but with management of the process conditions it would be possible maintain inhospitable conditions to methanogens and to achieve constant production of VFAs in a continuous fermentation process.

## Figures and Tables

**Table 1:** Characteristics of BW and inocula used. Ten-day test results reported previously in [11]. Biowaste (BW), volatile solids (VS), total solids (TS), total volatile fatty acids (VFAtot), soluble chemical oxygen demand (SCOD).

Substrates	Added VS (gVS/Bottle)	TS (%)	VS (%)	pH	VFAtot (g/L)	SCOD (g/L)
**28-day experiment**						
BW	4.79	28.97	26.88	4.24	2.32	91.24
Inoculum, control	9.59	4.85	2.74	7.77	0.24	3.27
Inoculum, thermal	9.59	6.96	3.78	9.56	0.28	8.35
Inoculum, freeze-thaw	9.59	5.50	3.02	8.56	0.26	3.91
**10-day experiment**						
BW	4.97	30.38	28.10	n.a.	2.50	120.35
Inoculum, control	9.95	6.04	3.75	n.a.	0.10	4.57
Inoculum, thermal	9.95	7.61	4.64	n.a.	0.20	11.21

n.a., not available.

## Supplementary Materials

The following are available online at https://www.mdpi.com/2076-2607/8/4/581/s1.
