# Supplementary material for: Effect of Inoculum Pretreatment on the Composition of Microbial Communities in Anaerobic Digesters Producing Volatile Fatty Acids
Source: Microorganisms. 2020 Apr 17;8(4):581. doi: 10.3390/microorganisms8040581 (PMC7232380; doi:10.3390/microorganisms8040581)
Supplement: Supplementary file 1 [file microorganisms-08-00581-s001.zip › SUPPLEMENTARY MATERIAL S1_Revised.docx]

**SUPPLEMENTARY MATERIAL S1**

**Effect of inoculum pretreatment on the composition of microbial communities in anaerobic digesters producing volatile fatty acids.**

Lucia Blasco^1^, Minna Kahala^1^, Elina Tampio^2^^[[1]](#footnote-1)^, Markku Vainio^1^, Satu Ervasti^3^, Saija Rasi^4^

*^1^Natural Resources Institute Finland (Luke), Production Systems, Tietotie 4, FI-31600, Jokioinen, Finland*

*^2^Natural Resources Institute Finland (Luke), Production Systems, Maarintie 6, FI-02150 Espoo, Finland*

*^3^Natural Resources Institute Finland (Luke), Production Systems, Ounasjoentie 6, FI-96200, Rovaniemi, Finland*

*^4^Natural Resources Institute Finland (Luke), Production Systems, Survontie 9A, FI-40500, Jyväskylä, Finland*

**Table S1.1.** pH, SCOD (soluble chemical oxygen demand) and VFAs (volatile fatty acids) in all tested days. Results are averages from triplicate analyses expressed with the standard deviation. The highest VFA production values are bolded. Test bottles with BW and control inoculum (CR), BW and thermally treated inoculum (TH) and BW and freeze-thaw treated inoculum (FR). 10-day results are reported previously in [11].

**Table S1.2.** The maximum methane production potential during the tests, methane from the inoculum subtracted. Test bottles with BW and control inoculum (CR), BW and thermally treated inoculum (TH) and BW and freeze-thaw treated inoculum (FR).

**Table S1.3.** The share of each VFA (% of the total VFA concentrations) during the 28 and 10-day fermentation tests. Test bottles with BW and control inoculum (CR), BW and thermally treated inoculum (TH) and BW and freeze-thaw treated inoculum (FR).

**Table S1.4.** Two- way PERMANOVA using Bray-Curtis index and 9999 permutations test results. Taxa significantly affected by treatment are labeled as T, or TD if Treatment or interaction of treatment and day was significant. The small letter at the beginning of each bacterial taxon name identifies the deepest classification level to which the OTUs were assigned. Inoculum treatment effect represents significant (*P* < 0.01) microbial abundance changes due to treatment itself (T) or interaction (TD). Control inoculum (CR), thermally treated inoculum (TH) and freeze-thaw treated inoculum (FR).

**Figure S1.1.** Rarefaction curves showed that the bacterial and archaeal communities.

**Figure S1.2.** The unweighted pair-group method with arithmetic mean (UPGMA) of the relationships between the community structure of archaea and bacteria. **a)** 28-day experiment **b)** 10-day experiment. The numbers are the sampling days and the replicates indicated A, B, C. Green color refers to the CR samples with BW and control inoculum, red color to TH samples with BW and inoculum thermal treatment, and blue color to FR samples with BW and inoculum freeze-thaw treatment.

**Table S1.1.** pH, SCOD (soluble chemical oxygen demand) and VFAs (volatile fatty acids) in all tested days. Results are averages from triplicate analyses expressed with the standard deviation. The highest VFA production values are bolded. Test bottles with BW and control inoculum (CR), BW and thermally treated inoculum (TH) and BW and freeze-thaw treated inoculum (FR). 10-day results are reported previously in [11].

|  | **pH** | **StDev** | **SCOD (g/l)** | **StDev** | **VFAtot (g/l)** | **StDev** | **VFAtot (gCOD/l)** | **StDev** |
| --- | --- | --- | --- | --- | --- | --- | --- | --- |
| **28-day experiment** | | | | | | | | |
| CR + BW | | | | | | | | |
| Day 0 | 7.54 | n.a. | 6.93 | n.a. | **0.30** | n.a. | n.a. | n.a. |
| Day 7 | 8.09 | 0.02 | 3.41 | 0.03 | 0.09 | 0.02 | 0.10 | 0 |
| Day 14 | 8.04 | 0.01 | 2.84 | 0.14 | 0.10 | 0.01 | **0.20** | 0 |
| Day 21 | 8.20 | 0.03 | 3.48 | 0.30 | 0.10 | 0.01 | **0.20** | 0 |
| Day 28 | n.m. | n.a. | 2.87 | 0.12 | 0.13 | 0.01 | **0.20** | 0 |
| TH + BW | | | | | | | | |
| Day 0 | 9.19 | n.a. | 9.36 | n.a. | 0.28 | n.a. | n.a. | n.a. |
| Day 7 | 6.88 | 0.11 | 12.94 | 0.27 | **8.96** | 0.4 | **11.5** | 0.6 |
| Day 14 | 7.87 | 0.11 | 7.03 | 1.28 | 2.70 | 1.38 | 3.9 | 1.7 |
| Day 21 | 7.97 | 0.04 | 6.09 | 0.66 | 2.21 | 0.58 | 3.1 | 0.8 |
| Day 28 | n.m. | n.a. | 3.63 | 0.21 | 0.31 | 0.15 | 0.4 | 0.2 |
| FR + BW | | | | | | | | |
| Day 0 | 7.68 | n.a. | 7.18 | n.a. | 0.31 | n.a. | n.a. | n.a. |
| Day 7 | 7.98 | 0.04 | 9.57 | 1.34 | **5.31** | 0.82 | **7.3** | 0.9 |
| Day 14 | 8.09 | 0.01 | 5.92 | 0.15 | 1.60 | 0.02 | 2.3 | 0 |
| Day 21 | 8.29 | 0.03 | 3.89 | 0.19 | 0.08 | 0.01 | 0.1 | 0 |
| Day 28 | n.m. | n.a. | 3.42 | 0.16 | 0.08 | 0 | 0.1 | 0 |
| 10-day experiment | | | | | | | | |
| CR+BW | | | | | | | | |
| Day 0 | 7.69 | n.a. | 8.35 | n.a. | 0.18 | n.a. | n.a. | n.a. |
| Day 1 | 7.34 | 0.01 | 5.84 | 0.12 | **2.90** | 0.61 | **3.6** | 0.8 |
| Day 3 | 7.65 | 0.03 | 3.59 | 0.07 | 0.00 | 0 | 0 | 0 |
| Day 6 | 7.63 | 0.03 | 3.13 | 0.05 | 0.07 | 0.06 | 0.1 | 0.1 |
| Day10 | 7.68 | 0.01 | 3.10 | 0.17 | 0.03 | 0.06 | 0.00 | 0.1 |
| TH + BW | | | | | | | | |
| Day 0 | 9.30 | n.a. | 11.33 | n.a. | 0.22 | n.a. | n.a. | n.a. |
| Day 1 | 6.47 | 0.12 | 8.53 | 1.34 | 2.63 | 0.51 | 3.2 | 0.8 |
| Day 3 | 5.81 | 0.02 | 11.50 | 1.69 | 7.00 | 0.17 | 8.5 | 0.2 |
| Day 6 | 5.72 | 0.01 | 11.91 | 0.86 | **8.20** | 0.7 | 10.4 | 1.1 |
| Day10 | 6.00 | 0.13 | 12.53 | 1.16 | 8.17 | 0.67 | **10.8** | 1 |
| n.m., not measured; n.a., not available |  |  |  |  |  |  |  |  |

**Table S1.2.** The maximum methane production potential during the tests, methane from the inoculum subtracted. Test bottles with BW and control inoculum (CR), BW and thermally treated inoculum (TH) and BW and freeze-thaw treated inoculum (FR).

|  | **CH4 (m3/tVS)^1^** |
| --- | --- |
| **28-day experiment** |  |
| CR+BW | 430.2 ± 13.5 |
| TH+BW | 563.4 ± 56.6 |
| FR+BW | 461.7 ± 11.7 |
| **10-day experiment^1^** |  |
| CR+BW | 435.4 ± 3.5 |
| TH+BW | 47.7 ± 1.4 |
| ^1^All results from day 28 / day 10. CH_4_ production from inoculum in 28-day experiment: CR: 62.7 ± 0.7 m^3^/tVS, TH: 5.0 ± 4.7 m^3^/tVS, FR: 88.8 ± 3.6 m^3^/tVS. CH4 production from inoculum in 10-day experiment: CR: 55.1 ± 1.1 m^3^/tVS; TH: 0 ± 0 m^3^/tVS | |

**Table S1.3.** Share of each VFA (% of the total VFA concentrations) during the 28 and 10-day fermentation tests. Test bottles with BW and control inoculum (CR), BW and thermally treated inoculum (TH) and BW and freeze-thaw treated inoculum (FR).

| % | **Acetic acid (%VFAtot)** | **Propionic acid (%VFAtot)** | **Butyric acid (%VFAtot)** | **Iso-butyric acid (%VFAtot)** | **Valeric acid (%VFAtot)** | **Iso-valeric acid (%VFAtot)** | **Caproic acid (%VFAtot)** |
| --- | --- | --- | --- | --- | --- | --- | --- |
| **28-day experiment** | | | | | | | |
| CR+BW | | | | | | | |
| Day 7 | 19 | 38 | 12 | 0 | 4 | 4 | 23 |
| Day 14 | 20 | 30 | 10 | 0 | 10 | 10 | 20 |
| Day 21 | 10 | 31 | 10 | 0 | 17 | 10 | 21 |
| Day 28 | 27 | 16 | 11 | 8 | 13 | 11 | 16 |
| TH + BW | | | | | | | |
| Day 7 | 70 | 9 | 14 | 2 | 2 | 3 | 0 |
| Day 14 | 40 | 35 | 3 | 8 | 2 | 10 | 1 |
| Day 21 | 44 | 44 | 1 | 2 | 1 | 8 | 1 |
| Day 28 | 73 | 10 | 4 | 2 | 1 | 5 | 4 |
| FR+BW | | | | | | | |
| Day 7 | 50 | 27 | 11 | 4 | 1 | 6 | 1 |
| Day 14 | 29 | 67 | 1 | 1 | 1 | 1 | 0 |
| Day 21 | 46 | 38 | 13 | 0 | 0 | 0 | 4 |
| Day 28 | 50 | 38 | 13 | 0 | 0 | 0 | 0 |
| **10-day experiment** | | | | | | | |
| CR+BW | | | | | | | |
| Day 1 | 70 | 22 | 2 | 2 | 0 | 3 | 0 |
| Day 3 | 0 | 0 | 0 | 0 | 0 | 0 | 0 |
| Day 6 | 100 | 0 | 0 | 0 | 0 | 0 | 0 |
| Day 10 | 100 | 0 | 0 | 0 | 0 | 0 | 0 |
| TH + BW | | | | | | | |
| Day 1 | 82 | 3 | 10 | 1 | 0 | 4 | 0 |
| Day 3 | 80 | 3 | 13 | 1 | 0 | 3 | 0 |
| Day 6 | 73 | 4 | 16 | 2 | 0 | 3 | 2 |
| Day 10 | 66 | 5 | 20 | 2 | 2 | 4 | 1 |

**Table S1.4.** Two- way PERMANOVA using Bray-Curtis index and 9999 permutations test results. Taxa significantly affected by treatment are labeled as T, or TD if Treatment or interaction of treatment and day was significant. The small letter at the beginning of each bacterial taxon name identifies the deepest classification level to which the OTUs were assigned. Inoculum treatment effect represents significant (*P* < 0.01) microbial abundance changes due to treatment itself (T) or interaction (TD). Control inoculum (CR), thermally treated inoculum (TH) and freeze-thaw treated inoculum (FR).

|  | **28-day experiment** | | | | **10-day experiment** | | |
| --- | --- | --- | --- | --- | --- | --- | --- |
|  | **Abundance (%) ± SD** | | |  | **Abundance (%) ± SD** | |  |
|  | **CR** | **FR** | **TH** | **p<0,01** | **CR** | **TH** | **p<0,01** |
|  |  |  |  |  |  |  |  |
| k_Archaea_unclassified | 1.50±1.90 | 0±0 | 0±0 | - | 1.78±1.37 | 0±0 | T, TD |
| o_pGrfC26_unclassified | 1.23±0.57 | 0.14±0.43 | 0.12±0.37 | T | 1.26±0.55 | 2.23±2.78 | - |
| f_Nitrososphaeraceae | 2.21±0.49 | 3.02±1.16 | 1.93±1.65 | - | 1.60±0.50 | 3.26±2.22 | - |
| f_Methanobacteriaceae | 10.43±1.13 | 13.51±1.70 | 15.06±8.46 | T | 10.52±2.11 | 35.72±5.64 | T |
| f_WSA2 | 3.40±1.42 | 1.63±0.71 | 1.64±1.42 | TD | 4.29±1.07 | 2.72±1.25 | T |
| c_Methanomicrobia_unclassified | 1.22±1.23 | 20.17±6.23 | 16.43±10.24 | T | 1.87±0.65 | 13.04±12.43 | T, TD |
| f_Methanocorpusculaceae |  | | |  | 0.20±0.40 | 0,54±1,08 | - |
| f_Methanomicrobiaceae | 12.30±3.84 | 12.58±5.17 | 36.03±11.35 | T | 11.93±3.12 | 7.31±1.91 | T |
| f_Methanoregulaceae | 1.59±0.74 | 0.48±0.79 | 0.98±0.88 | T | 0.47±0.44 | 0.58±0.96 | - |
| f_Methanospirillaceae | 12.01±0.63 | 3.91±1.72 | 2.14±2.10 | - | 12.7±2.69 | 6.90±5.51 | T |
| f_Methanosaetaceae | 37.24±5.87 | 22.64±8.5 | 5.62±4.11 | T | 37.08±4.34 | 12.58±3.12 | T |
| f_Methanosarcinaceae | 2.41±1.11 | 9.72±2.43 | 9.69±4.46 | - | 0.81±0.33 | 3.23±2.03 | TD |
| o_Methanosarcinales_unclassified | 1.66±0.92 | 1.93±1.52 | 0±0 | T | 3.03±1.19 | 0.28±0.55 | T, TD |
| o_YC-E6_unclassified | 0±0 | 0±0 | 0.87±1.50 | - | 0±0 | 2.81±4.00 | - |
| f_[Methanomassiliicoccaceae] | 11.97±2.81 | 10.11±2.43 | 8.99±2.86 | - | 11.39±2.73 | 8.1±5.62 | - |
| Others (<1%) | 0.68±0.71 | 0.13±0.4 | 0±0 | T | 1.10±0.80 | 0.72±1.11 | -- |
|  |  |  |  |  |  |  |  |
| k_Bacteria_unclassified | 11.56±0.73 | 9.44±0.73 | 9.22±0.97 | T | 11.78±0.83 | 6.78±1.48 | T |
| p_Actinobacteria | 6.00±1.00 | 5.56±0.53 | 5.78±1.09 | - | 4.11±0.78 | 9.44±1.01 | T, TD |
| p_Bacteroidetes | 16.11±1.36 | 15.67±1.58 | 2.56±2.07 | T | 17.44±0.88 | 2.56±0.53 | T, TD |
| p_Chloroflexi | 4.00±0.71 | 2.78±0.44 | 1.22±0.44 | T | 2.56±0.53 | 1.78±0.44 | T |
| p_Firmicutes | 34.67±1.8 | 45±1.32 | 69.11±4.46 | T | 36.44±1.33 | 63.67±1.73 | T |
| p_Proteobacteria | 8.56±0.73 | 5.11±0.78 | 5.00±0.87 | T, TD | 7.11±0.93 | 6.89±1.17 | - |
| p_Spirochaetes | 3.56±0.53 | 3.22±0.44 | 0±0 | T | 4.89±0.6 | 0.67±0.50 | T |
| p_Synergistetes | 2.56±0.53 | 2.11±0.33 | 2.33±0.5 | - | 2.11±0.33 | 2.00±0 | - |
| p_Tenericutes | 2.44±0.88 | 3.56±0.73 | 1.78±0.83 | T | 2.44±0.53 | 2.11±0.93 | TD |
| p_Verrucomicrobia | 1.04±0.25 | 0.41±0.23 | 0.16±0.15 | - | 1.33±0.50 | 0.11±0.33 | T |
| p_WWE1 | 1.44±0.53 | 1.56±0.53 | 0±0 | T, TD | 2.00±0.5 | 0.22±0.44 | T |
| Others (<2%) | 8.56±0.53 | 5.56±0.53 | 2.44±0.53 | T | 7.44±0.53 | 3.78±0.67 | T |


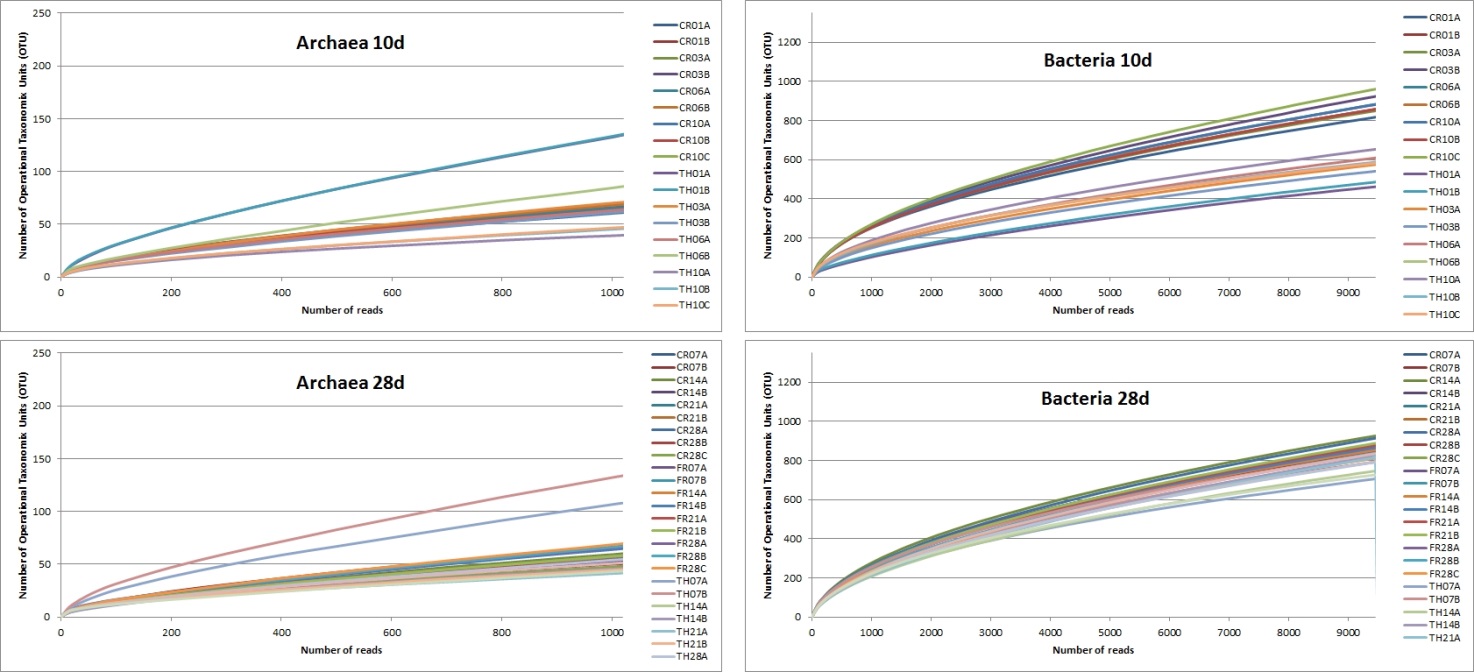


**Figure S1.1.** Rarefaction curves showed that the bacterial and archaeal communities.

**
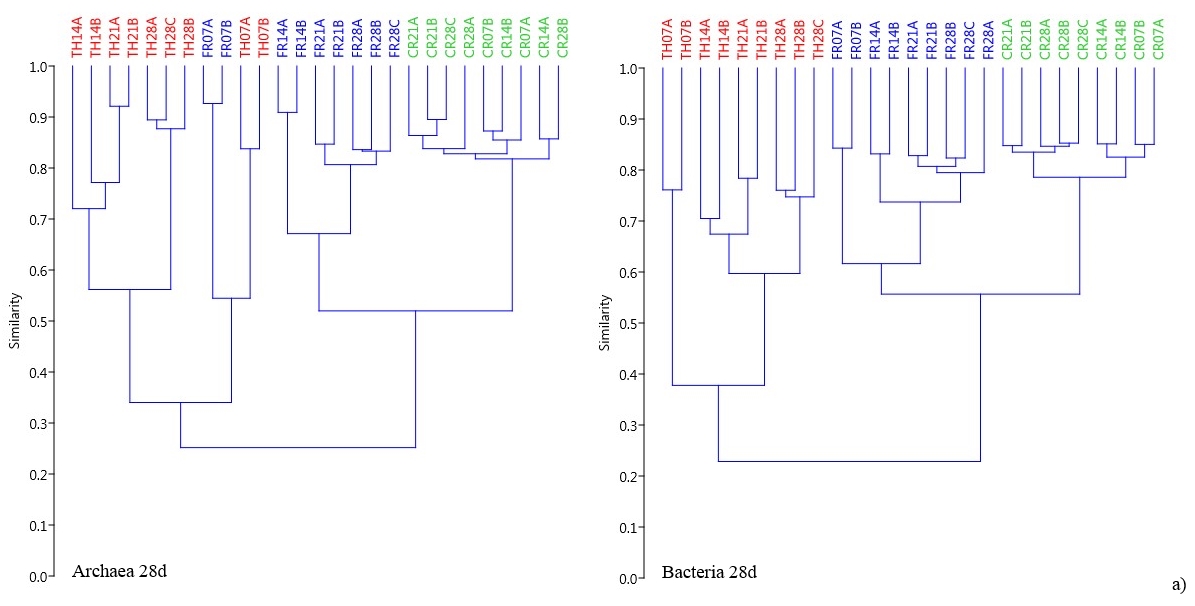

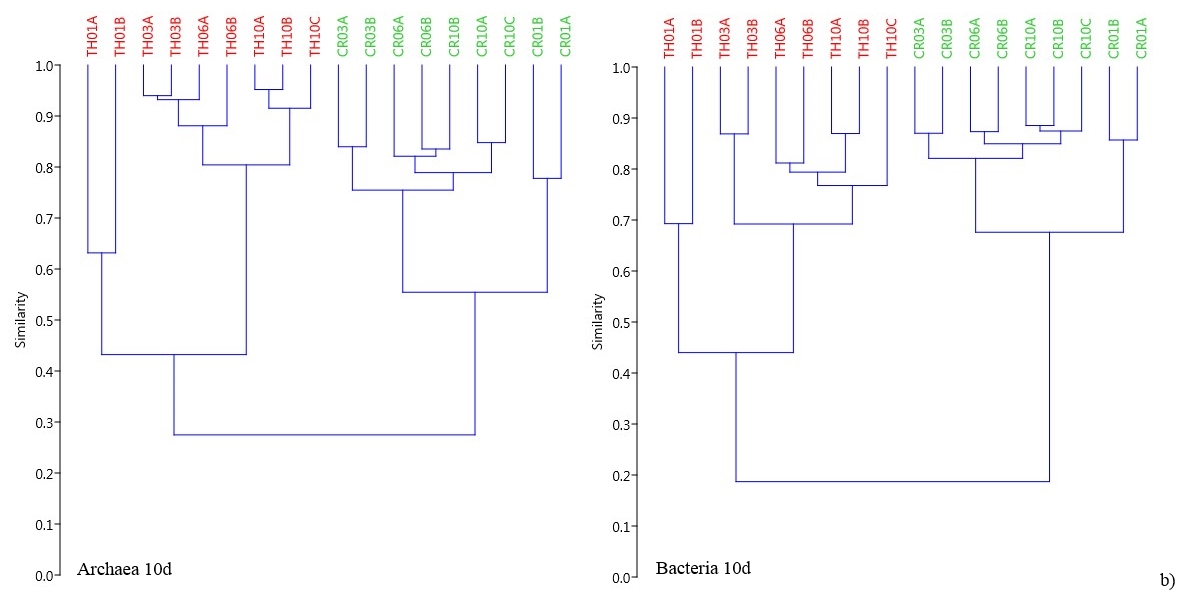
**

**Figure S1.1.** The unweighted pair-group method with arithmetic mean (UPGMA) of the relationships between the community structure of archaea and bacteria. **a)** 28-day experiment **b)** 10-day experiment. The numbers are the sampling days and the replicates indicated A, B, C. Green color refers to the CR samples with BW and control inoculum, red color to TH samples with BW and inoculum thermal treatment, and blue color to FR samples with BW and inoculum freeze-thaw treatment.

1. Corresponding author. tel.: +358 29 532 6573, e-mail address: elina.tampio@luke.fi [↑](#footnote-ref-1)
